# Fevers

**Published:** 1905-12-16

**Authors:** 


### FEVERS.

(*Continued from page* 167.)

**Plague.**—Plague still, unfortunately, prevails in India and no efforts which it seems possible to make there have succeeded in checking its ravages. Exceptional opportunities for its observation have been enjoyed by A. E. Elliot,[9] late Special Plague Officer to the Bombay Government, under whose personal notice, directly or indirectly, some 8,000 cases have come in the course of a few years. Elliot has at times observed undoubted motility in the bacillus, especially in the fulminating cases. He agrees with Bitter in placing it among the "septicæmic group." The characteristics of organisms belonging to this group are that when inoculated into a highly susceptible animal they pass at once into the blood-stream without the development of a local lesion, multiply rapidly, and produce a septicæmia. In less susceptible animals they require some foci in which to develop, and the elaborated toxins enter the blood, the bacteria themselves not entering the general blood-stream until shortly before death. A toxæmia, or sapræmia, is thus produced rather than a septicæmia. The less susceptible the animal, that is, the stronger the counteractive properties its tissues possess, the milder the local reaction and the milder the toxæmia, until in insusceptible or immune animals the local reaction and the toxæmia are reduced to *nil*. Rats—of which some species, at least, are highly susceptible to plague infection—die of a virulent septicæmia without local lesion. Elliot concludes that in man there are two degrees of susceptibility, depending mainly on the source of infection. The higher grade produces the pneumonic and septicæmic forms of plague. The lesser grade produces the bubonic form characterised by local reaction and toxæmia, but the local reaction is not, as is usual in septicæmic infections, at the seat of inoculation but in the nearest corresponding group of lymphatic glands. Plague specially involves the lymphatic system, and, however rapid the case, one or other group of glands is certain to be infected. Elliot has seen several cats suffering from buboes, but he is unable to confirm the opinion of Simpson, of Hong Kong, that poultry and pigeons suffer from the disease. In his hands repeated inoculation experiments on fowls and pigeons gave entirely negative results. In studying the incidence of plague in the lower animals the occurrence of other septicæmic diseases among them should be remembered—such as fowl, hog, and duck cholera, swine plague, and rabbit septicæmia—the specific organisms of which more or less closely resemble plague bacilli. It is possible the virus of plague has a saprophytic as well as a parasitic form. One experiment made by Elliot on the floor of a plague-infected hut at least suggests this. There are three channels of infection by which the bacilli may enter the body: the skin and mucous membranes, the alimentary canal, and the respiratory organs, and infection by these routes gives rise respectively to the bubonic, alimentary, and respiratory types of the disease. Either one of these forms may assume the features of a septicæmia. In bubonic plague Elliot finds that in men the inguinal, in women the axillary, and in children the cervical glands are most commonly affected, and he thinks these proclivities are explained by the habits, customs and practices of the native men, women, and children. If rats and guinea pigs are fed on plague-infected material they contract the disease, and it seems, therefore, likely that man may do so, and the post-mortem examination of certain cases in whom the only symptom was an acute and rapidly fatal diarrhœa, has revealed enlarged solitary and mesenteric glands containing the bacilli. Similarly, in the pneumonic form the bronchial and mediastinal glands are always infected. The alimentary and pneumonic forms are more fatal than the bubonic type, and of the last named, infection of the axillary and cervical glands is less often followed by recovery than involvement of the inguinal groups. Why is this? The explanation suggested is that the virulence of plague depends in a marked degree on the first group of glands which the virus has to pass through, entering the circulation much more rapidly where the meshwork of these is loose as in those of the intestine, than through compact encapsulated glands such as the inguinal. In regard to treatment the maintenance of the heart is the most important point to remember. In this connection also it is interesting to recall that in suppurating buboes, plague bacilli are not found. Is there some antagonism between staphylo- or strepto-cocci and plague bacilli, and, if so, could the encouragement of suppuration tend to mollify the disease? In a limited number of cases inoculations of antistreptococcic serum gave promising results, thirteen recovering out of twenty-one patients so treated. The observation that soldiers whose wounds were in a suppurating condition escaped infection with plague, was made as long ago as 1804 by the celebrated French surgeon Baron Larrey. Bruce Skinner[10] has studied the distribution of the various species of rats in the hope of throwing light on the infection of man by their agency. The Norway rat which is stronger and fiercer than the long-tailed variety and which has nearly driven the latter species out of Europe, did not appear in England until 1730, that is after the great plague epidemics had passed, hence it could not have assisted in their propagation. It is also said rarely to infest ships which are, however, peopled by the long-tailed variety. The question therefore is raised whether the Norway rat may be immune, and if so, whether its importation in order to keep down the susceptible long-tailed variety might not be feasible. Is it found in large numbers in the endemic plague areas which, according to Shadwell, include parts of Arabia, Mesopotamia, and Persia, parts of the North-West Provinces of India, Yumen in China, and East and Central Africa? An investigation of its distribution might have practical results, as would the precise determination as to whether or not it can be inoculated with plague.

[9] Lancet, June 10. [10] Brit. Med. Jour., May 6.

*(To be continued.)*

